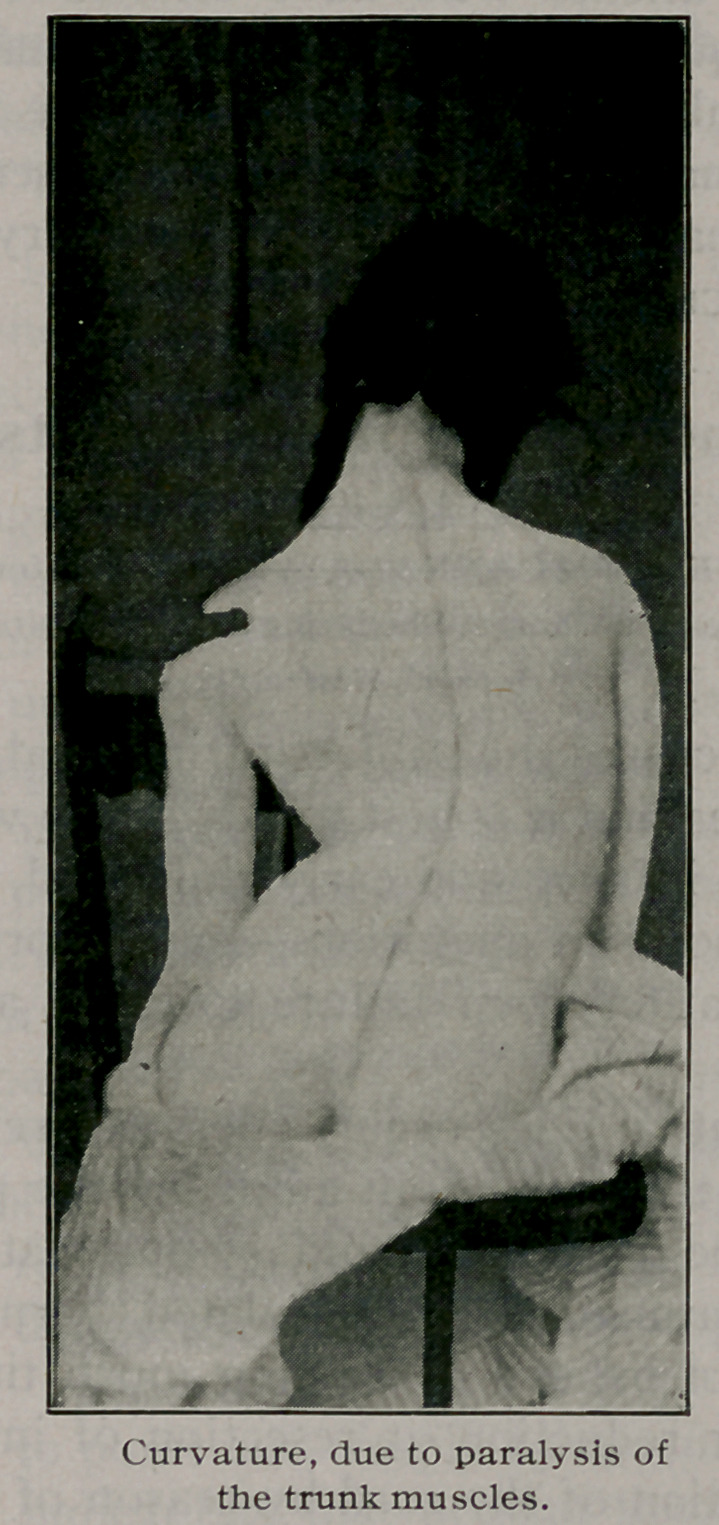# Some Comments on the Causes and Treatment of Lateral Curvature of the Spine

**Published:** 1907-11

**Authors:** Prescott Le Breton

**Affiliations:** Buffalo, N. Y.


### Some Comments on the Causes and Treatment of Lateral Curvature of the Spine.

By PRESCOTT LE BRETON, M. D., Buffalo, N. Y.

IN tabulating fifty private cases of spinal curvature, it was found that twenty-six were instances of the ordinary adolescent type, one was due to occupation, seven to various types of nervous disorders, one to empyema, two to Pott's disease, eight to unilateral hypertrophy or a short leg on one side, three to sciatica or sacroiliac affections, and two accompanied spondylitis de-

Ordinary adolescent type of severe grade.

formans. No cases caused by eye conditions, rickets, cervical rib, or congenital malformations were noted. This corresponds very well to the usual classifications and relative frequency of causes.

Lateral curvature is an acquired condition, due to the fact that the upright position is assumed. Fourfooted animals very rarely develop scoliosis. If a curvature once begins in the human being, gravity usually increases and continues the same, because the weight of the upper part of the body is now falling obliquely and tending to increase the distortion whenever the sitting or standing position is assumed. It is a pity that in a beginning case of the ordinary type we can not order for a period a fourfooted method of progression for several hours daily, the patient resting the remainder of the time. This is a means of treatment which would attack the cause directly and warrant a cure. Klapp attempts to imitate this in his system of exercises, that is, causing his patients to creep on hands and knees about the room for two hours daily, in this way exercising the spine and increasing its flexibility while it is relieved of strain and passively corrected. The hands and knees are protected by leather pads. Every step in this position calls upon the spine for considerable side flexion and many of the trunk muscles are brought into play.

Curvature caused by unilateral hypertrophy.

Ordinary adolescent type of mild grade.

There is a growing tendency among the profession to give up the complicated corrective exercises in the upright position and to persevere in symmetrical exercises in the prone posture,—in other words, developing tone and flexibility first and teaching the best standing position later on. Upon the presence of flexibility largely depends the prognosis in a given case. If we cannot correct and overcorrect the curve forcibly in a corrective machine, the best we can say to the patient is that we will try to prevent increase of curvature and hope to improve the case to a certain degree. The problem of correction of a rigid curve is still open. The conditions have been compared to the rigid flat foot. Lovett's and Wullstein's forcible correction in plaster-of-

Paris jackets gives the best promise at present. They apply successive jackets rapidly, each correcting more than the previous one, and the plaster is applied while the spine is held straight as far as possible in a machine. A certain degree of correction may be obtained in this way and a certain degree of relapse unfortunately follows as soon as the jackets are discontinued. Hoke has recently brought forward an added feature to this method. After the application of such a jacket the plaster is cut away on the concave and depressed side, and forcible breathing exercises are used to force the ribs outward and increase the amount of correction already obtained.

Marked curvature, due to sciatica.

Kyphosis and scoliosis, due to spondylitis deformans.

The keynote of treatment of any deformity is to overcorrect it and to hold it overcorrected until nature and the adoption of new habits establish a cure. The inability to follow out this rule in the case of curvature explains the poor results obtained. One may often accomplish a great deal by restoring symmetry and inducing new habits. In other words, examine the patient for flat foot, knock knee, unequal lower extremities, sloping of the pelvis to one side or swaying of the trunk to one side. By adding one half or three quarters of an inch to the heel of one shoe, by causing the patient to sit on an inclined chair, by calisthenics, by the formation of new habits in standing, sitting, or sleeping and by the adoption of the "West Point" attitude, we are treating the patient and indirectly treating the curvature. In eighteen of the twenty-six ordinary types of cases above mentioned, it was found that a change in the sole of the shoe affected the curvature favorably, especially if it were in the lumbar region. In fact, for several reasons, the writer has adopted the general rule that the higher the curve in the spine, the more difficult and uncertain the treatment.

One warning of especial significance should have stress laid upon it, which is this: the worst treatment possible for an early ordinary case is to advise a visit to the instrument maker, who puts on a support and gives no exercises. The patient, already suffering from poor muscular development, lack of tone, and bad postural habits, learns to depend on the artificial support which,

Deformity, due to empyema in early childhood.

of course, does not correct the curvature, and in a short space of time gravity has effected irreparable damage. Far better send the case out in the open air to indulge freely in games and sports of all kinds. Here is the explanation why, although mild grades of curvature before puberty occur as frequently in boys as in girls, later on we find such a large percentage of girls under treatment. They stopped play to become young ladies and a certain number soon must pay the penalty. It is true that girls should not indulge as actively as boys in games but a middle line may be pursued greatly to the benefit of the girl. Light exercises and play will preserve natural suppleness, freedom of joint motion, coördination and sense of balance. Three of the worst cases that the writer has seen, two of them recently coming under observation, were at first treated solely by support, during which period the opportunity for cure was lost, and marked bony deformity appeared.

In curvature due to occupation an opposite factor is at work, that is overwork for one side of the body. The case mentioned was that of a young man accustomed for several years to swing a sledge hammer to the right all day long. The one sided character of the work induced by degrees a very marked dorsal curve.

In cases due to nervous disease when the trunk muscles are partly paralyzed on one side, the convexity of the curve is usually toward the sound side. Here two observations may be

Curvature, due to paralysis of the trunk muscles.

made. These cases, as a rule, do better with support than without, although other means of treatment should be continued vigorously until full growth is obtained. Also the patient must be seen frequently from the ages of twelve to sixteen because some of them at this time grow much worse. The writer has seen three cases of anterior poliomyelitis, in whom a slight curvature existed for several years, which increased most rapidly to a severe degree at the ages mentioned. At such a time there should be employed daily massage, electricity, and forced breathing while one-half the chest is held by adhesive plaster or similar means.

Empyema, pleuropneumonia, or rib necrosis, may induce a depression of the chest on one side, which may be followed by rotation and curvature. After the deformity has become fixed by bony and ligamentous changes breathing exercises can not alter it. Support is often indicated to relieve backache and discomfort.

Occasionally in Pott's disease scoliosis becomes an added factor. A plaster jacket applied while both deformities are corrected as far as possible, offers the best means of treatment. The same may be said of the curvature accompanying spondylitis deformans. Patients who have sciatica or disease of the sacroiliac joint frequently lean away from the affected side until a lumbar curve results. Not much can be done for the curvature itself until the primary condition is cured, when a high heel on the opposite side may help to restore symmetry.

23 Irving Place.

## Figures and Tables

**Figure f1:**
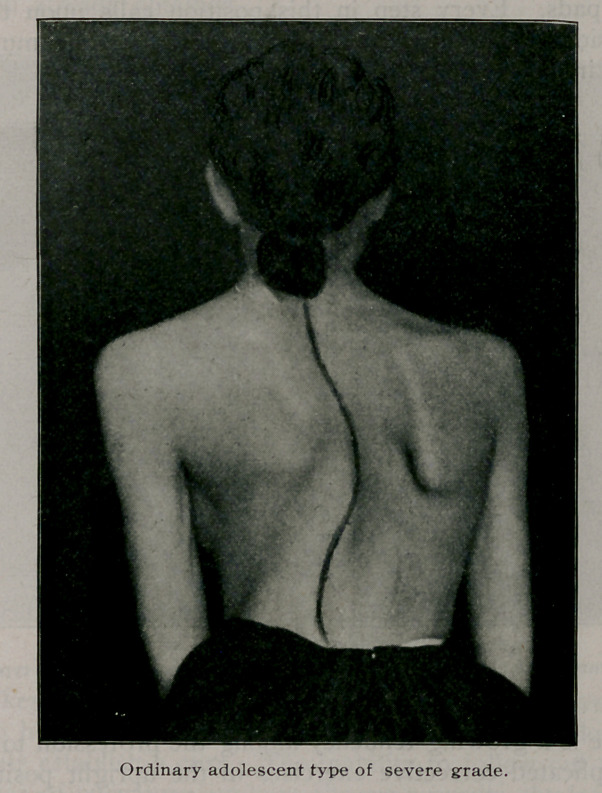


**Figure f2:**
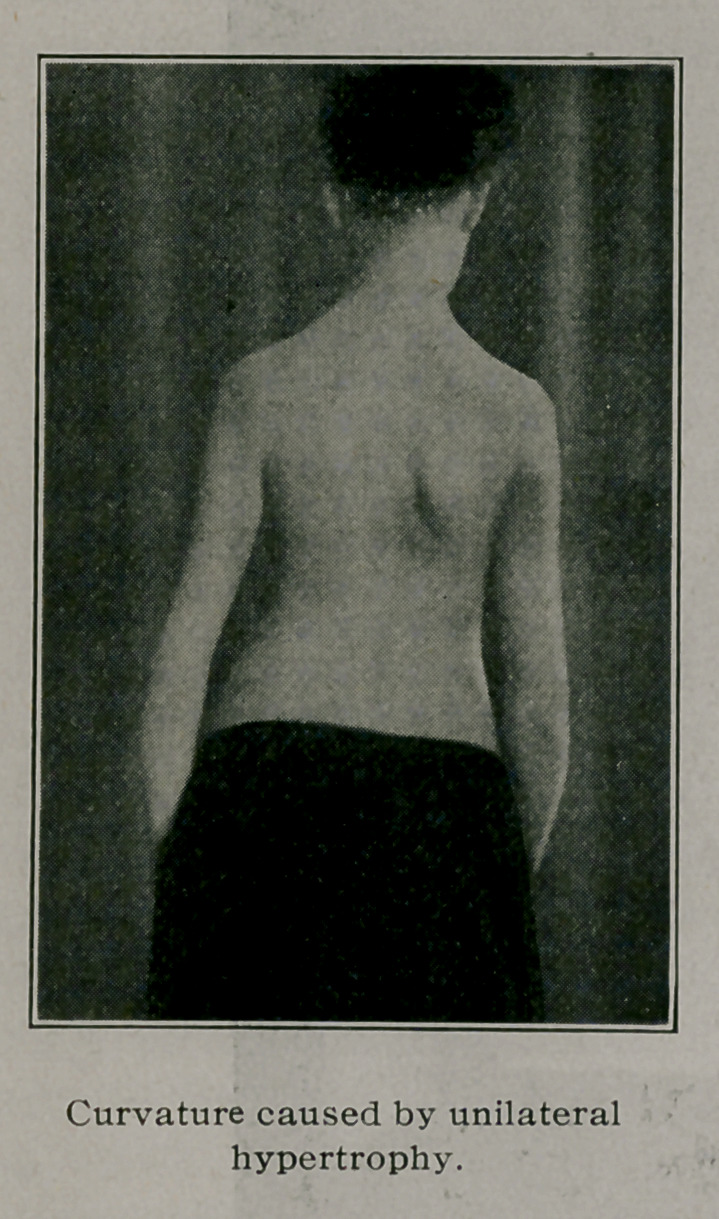


**Figure f3:**
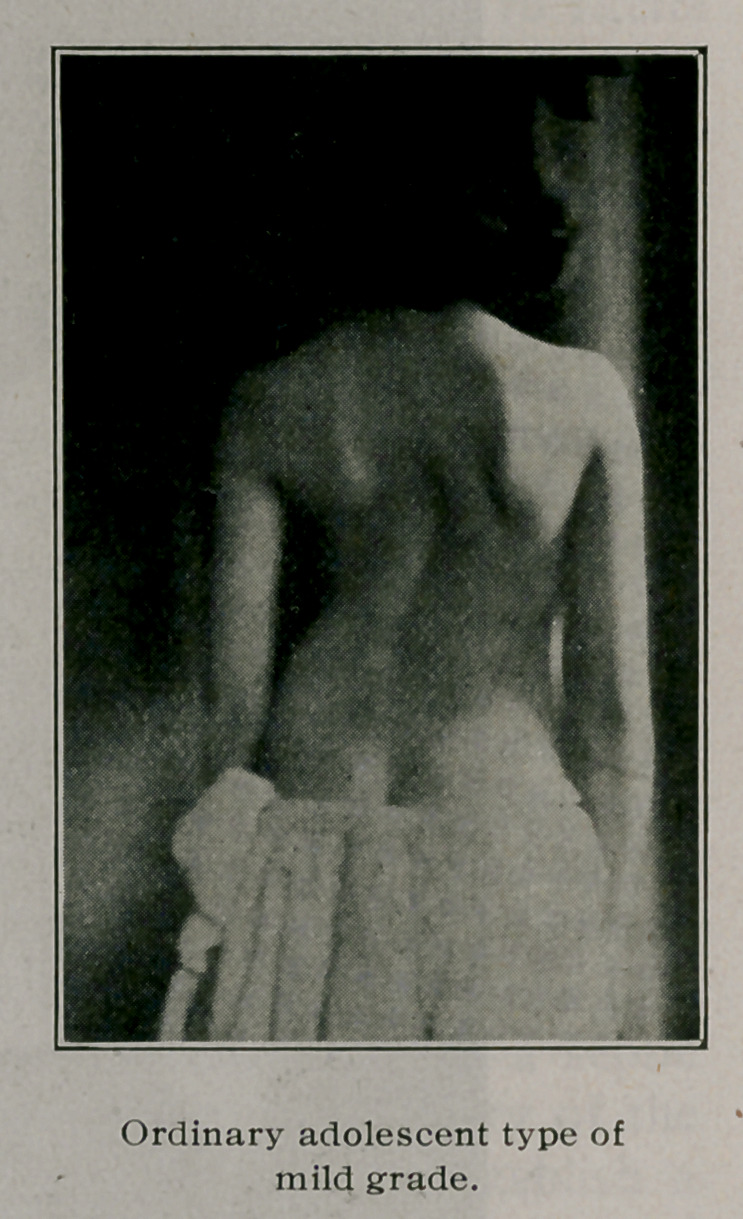


**Figure f4:**
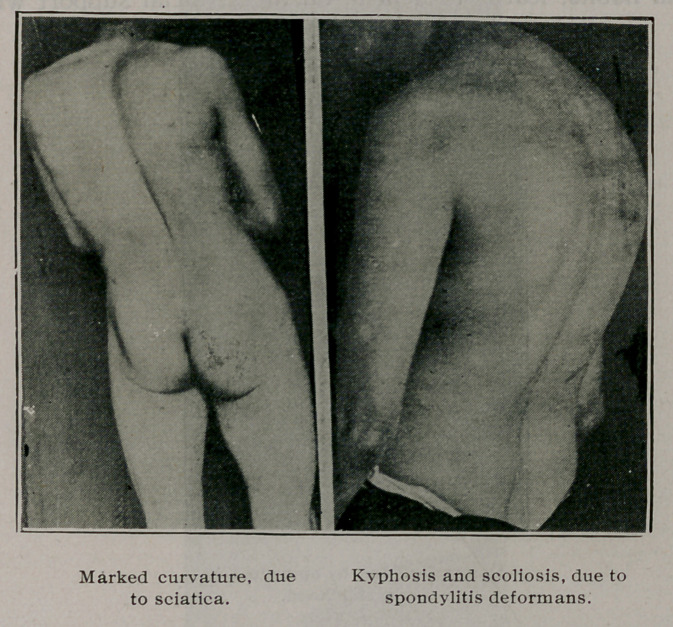


**Figure f5:**
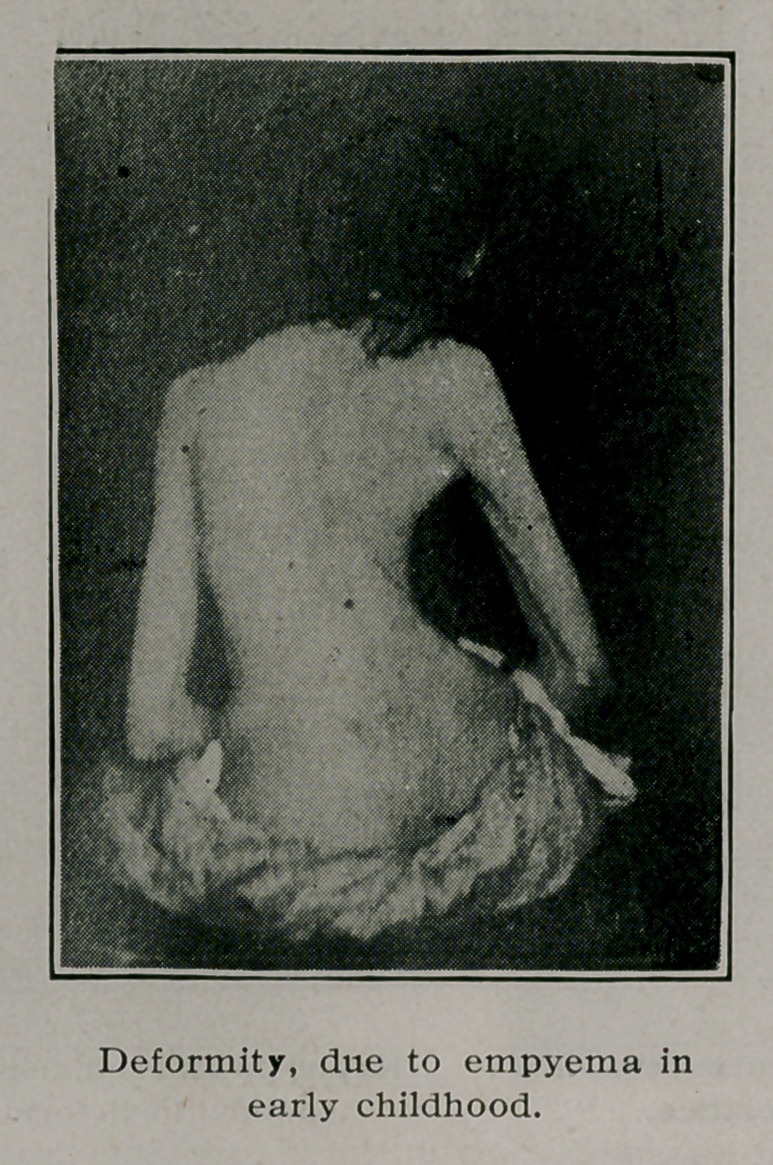


**Figure f6:**